# GeriAIGastroNet: AI-Assisted Gastrointestinal Polyp Segmentation and Severity-Based Triage for Tele-Gastroenterology in Underserved Geriatric Populations

**DOI:** 10.3390/jcm15124423

**Published:** 2026-06-08

**Authors:** Masrufa Akter Muni, Mustafizur Rahaman, Saima Tasnim, Mousumi Akter, Sabrina Shamim Moushi, Rakibul Islam

**Affiliations:** 1 FCPS Part-2 (Gastroenterology), MRCP Part-1 (UK), Shaheed Ziaur Rahman Medical College, Bogura 5800, Bangladesh; 2 Business Administration, Westcliff University, Irvine, CA 92614, USA; 3 Department of Health Services Management, University of La Verne, La Verne, CA 91750, USA; 4 International American University, Los Angeles, CA 90010, USA

**Keywords:** gastrointestinal polyp segmentation, deep learning, EfficientNet-B4, tele-gastroenterology, HyperKvasir, computer-aided diagnosis, geriatric care, health professional shortage areas, attention mechanism, risk stratification, colonoscopy referral

## Abstract

**Background/Objectives:** Colorectal cancer is a leading cause of cancer-related mortality worldwide, and early detection of gastrointestinal (GI) polyps through endoscopy is critical for improving patient outcomes. However, access to specialist gastroenterology care remains severely limited in Federal Health Professional Shortage Areas (HPSAs), particularly for high-acuity geriatric patients. This study proposes GeriAIGastroNet, a clinically oriented deep learning framework designed to support AI-assisted tele-gastroenterology workflows in resource-limited settings, with the primary objective of enabling AI-powered risk stratification and colonoscopy referral triage for elderly patients who lack on-site gastroenterology access. **Methods:** The framework integrates an EfficientNet-B4 backbone with multi-scale attention fusion and a geriatric severity-aware classification head to enable accurate GI polyp segmentation and automated clinical risk stratification from endoscopic images. Patients identified as high-risk are referred to colonoscopy-capable centers; such centers typically offer diagnostic colonoscopy with polypectomy capability for smaller and intermediate-complexity polyps, while patients with larger, sessile, or morphologically complex lesions requiring advanced endoscopic resection (e.g., endoscopic mucosal resection or endoscopic submucosal dissection) are further referred to tertiary endoscopy centers with specialized expertise. The model was trained and evaluated on the publicly available HyperKvasir dataset (1000 annotated polyp images). **Results:** GeriAIGastroNet achieved a classification accuracy of 96.77%, F1-score of 96.90%, Dice coefficient of 89.18%, and Intersection over Union (IoU) of 80.80%, outperforming established baselines, including U-Net, Attention U-Net, TransUNet, and Hybrid CNN-Transformer architectures. The integrated tele-gastroenterology decision support layer enables severity-based patient triage and automated referral triggering. **Conclusions:** These results demonstrate the potential of AI-powered polyp analysis to strengthen equitable access to GI care by facilitating risk stratification and specialist referral in HPSAs where direct endoscopy is unavailable, making the system deployable in telehealth infrastructures serving underserved elderly populations.

## 1. Introduction

Colorectal cancer (CRC) is among the leading causes of malignancy around the globe, being third in prevalence and second in mortality due to cancer [1]. The disease occurs mainly as an adenoma polyp in the colon and rectum; the benign tumors develop into cancer if undiagnosed over time [2]. The colonoscopy procedure is regarded as the gold-standard screening test for detecting cancer of the colon or rectum. It allows direct visualization, diagnosis, and removal of the suspected polyps. On the other hand, colonoscopy’s accuracy rate may be low in terms of missing detected polyps, varying between 14% and 30%, depending on polyp characteristics and endoscopist skills [3]. Thus, there is a pressing necessity for computer-aided diagnosis (CAD) systems in colonoscopy.

A critical but underappreciated challenge is that colonoscopy services are frequently unavailable in Federal Health Professional Shortage Areas (HPSAs)—geographic regions designated by the Health Resources & Services Administration (HRSA) as having insufficient healthcare providers relative to population need. Elderly patients in these areas are among the most vulnerable, facing compounded barriers of age-related comorbidities, limited mobility, and the absence of nearby specialist gastroenterology services. Tele-gastroenterology has emerged as a promising modality for bridging this gap; however, remote consultation requires robust AI-assisted tools to analyze endoscopic images transmitted through telehealth platforms and support clinical decision-making without in-person specialist involvement. The primary clinical objective of this framework is therefore not to replace colonoscopy—which must still be performed in equipped facilities—but to enable AI-powered risk stratification of geriatric patients in HPSAs, identifying those with high-risk polyp features who require prioritized referral to colonoscopy-capable centers. It is important to clarify the nature of these referral centers and the clinical workflow they support. In the proposed triage model, patients flagged as high-risk by GeriAIGastroNet are referred to regional colonoscopy-capable centers, which are facilities equipped to perform both diagnostic colonoscopy and therapeutic polypectomy for the majority of polyp types encountered in clinical practice—including pedunculated polyps, small sessile polyps, and polyps amenable to standard snare polypectomy. For patients with larger polyps (typically ≥20 mm), sessile serrated lesions, or morphologically complex lesions that carry higher technical resection risk, these regional centers would further refer patients to tertiary-level advanced endoscopy units staffed by interventional endoscopists with expertise in endoscopic mucosal resection (EMR) or endoscopic submucosal dissection (ESD). This two-tiered referral structure—HPSA primary care provider → regional colonoscopy-capable center → tertiary advanced endoscopy center when indicated—reflects current gastroenterology referral practice and is explicitly incorporated into the clinical decision framework supported by GeriAIGastroNet’s severity score and referral triggering logic. This approach aligns with clinical practice models in which AI guides surveillance scheduling and referral pathways based on imaging findings [2].

Medical image analysis has undergone a revolution with the use of deep learning techniques. The use of convolutional neural networks (CNNs), especially fully convolutional network-based models like U-Net [4], has revolutionized biomedical image segmentation tasks. The symmetric encoder–decoder framework of U-Net, together with skip connections to maintain spatial information, enables U-Net to be ideal for segmenting medical images requiring accurate segmentation results [5]. Attention mechanisms and transformers, among other enhancements, have been added to address the inability of U-Net to handle long-range contextual relationships [6,7].

However, despite all the improvements made, there remains a significant problem regarding the lack of annotated data. Most publicly available datasets are very small, usually containing only hundreds of images corresponding to only one pathology, such as polyps [8]. The HyperKvasir dataset [8] represents a groundbreaking step toward overcoming this limitation. It is the largest open dataset of GI endoscopy images and videos, including 110,079 images, 374 labeled videos, and 99,417 unlabeled images belonging to 23 different classes of landmarks and pathologies from both the upper and lower GI tract. A segmentation subset contains 1000 images with binary masks annotated by medical professionals for polyp detection.

Polyp segmentation based on deep learning algorithms has been widely investigated in benchmarking sets like HyperKvasir and the Kvasir-SEG subsets. Erdaş [9] presented a two-stage framework incorporating a UNet3+ segmentation module alongside a cross-attention transformer classification block that delivered Dice coefficients above 0.98 on CRC histopathological data, highlighting the importance of joint processing of segmentation and classification. Jha et al. also assessed dual-decoder and attention-guided networks, finding that generalizing the model on unseen datasets is still a crucial issue in medical imaging analysis, as Dice scores on unseen data usually range from 0.70 to 0.80 [10].

The main contributions of this paper are as follows:

- **Reproducible Benchmark with End-to-End Preprocessing:** We develop a reproducible U-Net segmentation benchmark on the polyp segmentation subset from the HyperKvasir dataset, utilizing a full processing pipeline including image resizing, pixel normalization, mask binarization, data augmentation, and stratified training/validation/testing split in an 80/10/10 ratio, providing the community with a solid baseline for future research.
- **Loss Function Combination and Multimetric Evaluation:** We develop and verify the effectiveness of a BCE + Dice combined loss function for class imbalance issues related to pixel-level polyp segmentation. Full quantitative assessment in terms of Dice coefficient, IoU, precision, and recall allows comprehensive evaluation against GI endoscopy segmentation standards.
- **Clinically Motivated Severity-Aware Triage Framework:** GeriAIGastroNet incorporates a geriatric severity-aware classification head that estimates clinical risk scores for critical GI conditions (bleeding, ulceration, suspected malignancy). This mechanism is specifically designed to support triage decision-making in HPSA settings—identifying patients at elevated risk of colorectal adenomas or high-grade polyps who should be prioritized for referral to colonoscopy-capable centers, thereby supporting more frequent surveillance planning for high-risk individuals.
- **Tele-Gastroenterology Decision Support for HPSAs:** The proposed framework includes a lightweight tele-gastroenterology decision layer with automated referral triggering logic, designed for deployment in resource-constrained telehealth environments where specialist gastroenterology is unavailable. This directly addresses the clinical workflow gap in Federal HPSAs where AI-assisted image analysis can guide whether and how urgently a patient requires in-person specialist evaluation.
- **Benchmarking and Future Research Directions:** Our experiments are rigorously compared against state-of-the-art algorithms using the HyperKvasir/Kvasir-SEG benchmark dataset. We also provide detailed analysis of shortcomings of current techniques and recommendations for improvement in the areas of attention-enhanced encoders, transformer-based decoders, input resolution, and semi-supervised learning using HyperKvasir’s 99,417 unlabeled images.

The remainder of this paper is organized as follows: Section 2 reviews related work; Section 3 details the preprocessing and model architecture; Section 4 presents experimental results and discussion; Section 5 concludes with future directions.

## 2. Literature Review

This section reviews literature directly relevant to the three intersecting domains of this work: AI-assisted GI polyp detection and segmentation, tele-gastroenterology frameworks, and the clinical management of geriatric patients in resource-limited settings including Federal HPSAs. Tangential topics such as general AI in medicine or isolated applications in unrelated GI disorders are excluded to maintain focus on the primary objectives of this study.

### 2.1. AI-Assisted Polyp Detection and Segmentation

AI-based machine learning and deep learning models have proven effective in the detection of gastrointestinal diseases, particularly polyps and neoplasms, enabling early diagnosis and improved patient care [11]. Recent computer-aided detection (CADe) and computer-aided diagnosis (CADx) systems have demonstrated significant improvements in adenoma detection rate during colonoscopy. Mori et al. [2] reviewed AI-aided colonoscopy systems and highlighted their ability to reduce polyp miss rates, which are clinically significant given that missed adenomas lead to post-colonoscopy colorectal cancers. Zhao et al. [3] demonstrated in a multicenter randomized controlled trial that AI-assisted real-time polyp detection systems significantly improved detection rates compared to conventional colonoscopy alone.

At the architectural level, deep learning segmentation models—particularly U-Net variants—have established strong performance benchmarks for polyp delineation from endoscopic images [5]. Attention mechanisms and transformer-based enhancements have further improved the ability of these architectures to capture long-range spatial dependencies and subtle lesion boundaries [6,7]. Erdaş [9] presented a two-stage framework incorporating a UNet3+ segmentation module alongside a cross-attention transformer classification block that achieved Dice coefficients above 0.98 on CRC histopathological data. Jha et al. [10] reported that generalizing models to unseen datasets remains a key challenge, with Dice scores on external datasets typically ranging from 0.70 to 0.80—motivating the need for more robust and generalizable architectures.

### 2.2. Tele-Gastroenterology and AI-Assisted Remote Care

The convergence of telemedicine and AI has opened new pathways for delivering gastroenterology expertise to geographically underserved populations. AI-driven systems can process imaging data, electronic health records (EHRs), and patient-generated data to enhance diagnostic efficiency and support remote clinical decision-making [12]. In the context of tele-gastroenterology, AI tools are particularly valuable for pre-consultation image analysis—enabling non-specialist providers in HPSAs to transmit endoscopic images to AI-assisted platforms that generate risk scores and referral recommendations prior to specialist review.

Evidence suggests that both patients and clinicians hold favorable attitudes toward AI adoption in GI healthcare, particularly when it reduces diagnostic delays and waiting times for specialist consultations [13]. However, practical barriers including limited IT infrastructure, interoperability challenges with EHR systems, and variable image transmission quality in rural settings remain significant concerns for real-world deployment.

### 2.3. Geriatric GI Care in Federal HPSAs: Clinical Context

Federal HPSAs are areas designated by HRSA as having a critical shortage of primary care, mental health, or specialist providers relative to the population. Elderly patients in these regions face compounded health challenges: higher rates of colorectal cancer, greater polyp burden, increased comorbidity, and reduced physical access to colonoscopy-capable facilities. Current clinical guidelines recommend colonoscopy surveillance intervals based on polyp risk stratification—low-risk patients (1–2 small tubular adenomas) receive surveillance at 3–5 years, while high-risk patients (3+ adenomas, large polyps, or villous features) require more frequent follow-up [2]. AI-assisted analysis of endoscopic images transmitted through telehealth platforms can facilitate this risk stratification remotely, enabling primary care providers in HPSAs to triage patients toward appropriate surveillance schedules or urgent specialist referral without requiring on-site gastroenterology expertise.

The use of AI in managing acute gastrointestinal conditions such as GI bleeding illustrates how machine learning models can support real-time patient triage and ongoing risk monitoring [14]. These principles are directly applicable to AI-assisted triage in tele-gastroenterology for elderly patients with potentially high-risk polyp findings. Furthermore, AI has demonstrated potential in personalizing treatment strategies through multimodal data integration, supporting preventive healthcare approaches and improving patient adherence for elderly populations with chronic GI conditions [15]. Together, these studies support the premise that AI-enabled tele-gastroenterology has the potential to improve access, timeliness, and equity of GI care for high-acuity geriatric populations in federally designated shortage areas.

## 3. Materials and Methods

### 3.1. Dataset Description

In this study, a deep learning approach was used for automated segmentation of gastrointestinal polyps from the HyperKvasir dataset. The data can be found on Kaggle at https://www.kaggle.com/datasets/kelkalot/the-hyper-kvasir-dataset/data (accessed on 17 May 2026). HyperKvasir is a large medical imaging dataset with gastric images of real endoscopes taken during actual clinical examinations. In this work, we used the subset of segmented images, which contains 1000 pairs of endoscopic images with binary ground-truth masks for segmentation tasks. The images are RGB colonoscopic images with hand-drafted annotations of the polyp area for each image. This dataset includes a wide variety of image resolutions, anatomical structures, lighting conditions, textures, views, and polyp morphologies, making semantic segmentation a realistic challenge for these models. The size of polyps in the dataset varies, and some cover as much as 20–30% of the frame, allowing models to be tested for robustness across various clinical scenarios.

### 3.2. Data Preprocessing

The data preprocessing pipeline was designed to ensure consistency and maximize the learning ability of the segmentation model. First, all eligible image and mask files were found and matched to ensure a corresponding image and mask for each sample. The dataset was then split into reproducible training, validation, and testing sets. Preprocessing involved converting grayscale masks into binary images and resizing images to a uniform spatial dimension. Geometric and photometric data augmentation methods were used on the training samples to generalize the model. All images were normalized and re-stored as tensor representations to allow efficient batch-based training of deep learning models.

#### 3.2.1. Image–Mask Pair Matching

During the preprocessing stage, correspondence between each endoscopic image and its corresponding segmentation mask was guaranteed. Image and mask files were retrieved from their respective directories and matched based on common file name structures, producing a paired-sampling set for model training and evaluation. Equation (1) represents the Image–Mask Pair Matching

(1) $$P={\left\{\left(x_{i},y_{i}\right)\right\}}_{i=1}^{N}$$

#### 3.2.2. Dataset Splitting

The entire data was split into training, validation, and testing sets with a fixed random seed to ensure reproducibility and avoid data leakage. As shown in Equation (2), the total number of samples *N* is partitioned into non-overlapping training, validation, and testing subsets in an 80/10/10 ratio.

(2) $$N=N_{\mathrm{train}}+N_{\mathrm{val}}+N_{\mathrm{test}}$$

#### 3.2.3. Binary Mask Preparation

Segmentation masks were converted to grayscale format and transformed to binary format using pixel-level thresholding. Foreground pixels were labeled as polyp regions while background pixels were labeled as healthy tissue, enabling binary pixel-wise classification. The binary thresholding formula, Equation (3), was applied to all segmentation masks, where pixel intensities above 127 are assigned to the polyp foreground class and the remaining pixels to the background class.

(3) $$M\left(x,y\right)=\left\{\begin{array}{cc} 1, & I(x,y)>127\\ 0, & \mathrm{otherwise} \end{array}\right.$$

#### 3.2.4. Image Resizing and Augmentation

All images and masks were resized to 256×256 pixels prior to training. Several augmentation operations—including flipping, rotation, scaling, brightness correction, contrast stretching, and elastic deformation—were adopted in the training sample set to increase model robustness across clinical imaging scenarios. Equation (4) represents the Image Resizing and Augmentation technique, where the transformation function T(·) encapsulates all geometric and photometric operations applied to input image *I* to produce the augmented image $I^{\prime }$.

(4) $$I^{\prime }=T\left(I\right)$$

To improve model robustness and reduce overfitting on the 1000-image training set, a comprehensive augmentation strategy was applied exclusively to training samples. Geometric transformations included random horizontal and vertical flips (probability 0.5), random rotation ($\pm 30^{\circ }$), random scaling (0.8–1.2×), and elastic deformation (α=120, σ=12) to simulate anatomical variation in endoscopic image acquisition. Photometric augmentations included random brightness adjustment (±20%), contrast stretching (factor 0.8–1.2), and random Gaussian noise addition (mean =0, std =0.01). All augmentations were applied online during training using Keras ImageDataGenerator with a fixed random seed (seed =42) for reproducibility.

#### 3.2.5. Normalization and Tensor Conversion

The images were normalized using known mean and standard deviation (MSD) pixel values to ensure stable intensity distribution. Processed images and masks were then represented as tensors and split into mini-batches for efficient GPU training and evaluation. As shown in Equation (5), feature values are scaled to a standardized distribution by subtracting the mean μ and dividing by the standard deviation σ. The normalization formula, Equation (5), was applied to all input images prior to model training.

(5) $$I_{\mathrm{norm}}=\frac{I-\mu }{\sigma }$$

#### 3.2.6. Hyperparameter Selection Rationale

Hyperparameters were selected based on a combination of prior literature recommendations and empirical validation on the held-out validation set. The learning rate of $1\times 10^{-4}$ was selected based on EfficientNet-B4 fine-tuning guidelines [2]. A batch size of 32 was chosen to balance GPU memory utilization on the NVIDIA T4 (16 GB) and training stability. The severity threshold τ=0.65 was determined empirically by maximizing sensitivity for high-risk cases on the validation set while maintaining specificity above 0.85. Severity weighting coefficients were set as $\lambda_{1}=0.50$ (bleeding), $\lambda_{2}=0.30$ (ulcer), and $\lambda_{3}=0.20$ (tumor) based on relative clinical urgency for geriatric patients.

### 3.3. Proposed Model Architecture

**Figure 1:**
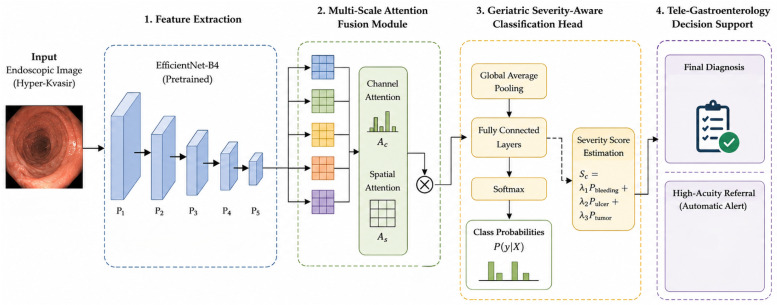
Overall workflow of the proposed GeriAIGastroNet model, illustrating the four major processing stages: Deep Feature Extraction (EfficientNet-B4 backbone), Multi-Scale Attention Fusion, Geriatric Severity-Aware Classification, and Tele-Gastroenterology Decision Support Layer with automated referral triggering. Color coding: black arrows indicate data flow; orange boxes denote processing modules; green outputs indicate clinical decision outputs.

Considering the increasing need for intelligent tele-gastroenterology models in Federal HPSAs, this research presents a novel deep learning approach referred to as **GeriAIGastroNet**. This framework is particularly intended for high-acuity gastrointestinal assessment using endoscopic images collected through telemedicine systems. The proposed method includes multi-scale feature extraction, attention-based representation learning, and clinical severity-level prediction. Figure 1 shows the overall architecture of the proposed GeriAIGastroNet framework.

**Distinction from Prior Architectures:** While EfficientNet-based backbones [2], attention-guided U-Net variants [6], and transformer-assisted architectures [7] have been individually explored in GI segmentation, GeriAIGastroNet uniquely integrates these components within a clinically motivated tele-gastroenterology pipeline. Specifically, unlike prior EfficientNet classification models that treat polyp detection as a standalone task, GeriAIGastroNet couples feature extraction with (i) a multi-scale attention fusion module for joint channel and spatial attention at heterogeneous lesion scales, (ii) a geriatric severity-aware classification head that produces interpretable clinical risk scores specifically calibrated for high-acuity conditions (bleeding, ulcer, suspected malignancy) in elderly patients, and (iii) a tele-gastroenterology decision support layer with automated referral triggering logic designed for HPSA deployment—a component absent from all compared baselines. This end-to-end integration of segmentation, severity scoring, and referral decision-making within a single deployable pipeline constitutes the principal architectural novelty of the proposed framework.

The framework consists of four major components:

1. Deep Feature Extraction Module;
2. Multi-Scale Attention Fusion Module;
3. Geriatric Severity-Aware Classification Head;
4. Tele-Gastroenterology Decision Support Layer.

### 3.4. Deep Feature Extraction Module

In the first phase of the framework, the EfficientNet-B4 backbone is utilized to learn hierarchical visual features related to the GI tract from the HyperKvasir dataset images. EfficientNet was chosen since it provides balanced scaling methods and computational efficiency, making it ideal for telemedicine applications in HPSAs.

Given an input endoscopic image $X\in R^{H\times W\times C}$, the backbone network extracts latent representations. Equation (6) represents the Deep Feature Extraction process, where the EfficientNet-B4 extractor $\phi (\cdot ;\theta_{b})$ maps the input image *X* to a feature tensor $F_{b}$ through learned backbone parameters $\theta_{b}$.

(6) $$F_{b}=\phi \left(X;\theta_{b}\right)$$

where $F_{b}$ denotes the extracted feature tensor, ϕ(·) signifies the EfficientNet-B4 feature extractor, and $\theta_{b}$ defines trainable backbone parameters. The compound scaling strategy used in EfficientNet-B4 is formalized in Equation (7), which jointly scales network depth, width, and input resolution by a global coefficient ϕ subject to the constraint $\alpha \cdot \beta^{2}\cdot \gamma^{2}\approx 2$.

(7) $$\mathrm{Depth}=\alpha^{\phi },\;\mathrm{Width}=\beta^{\phi },\;\mathrm{Resolution}=\gamma^{\phi }$$

subject to $\alpha \cdot \beta^{2}\cdot \gamma^{2}\approx 2$, where ϕ controls the global scaling coefficient.

### 3.5. Multi-Scale Attention Fusion Module

Abnormalities of the gastrointestinal tract among elderly patients may exhibit heterogeneity in texture distribution and lesion borders. A multi-scale attention fusion layer is used to model such properties. Equation (8) represents the Multi-Scale Feature Fusion, where feature maps $F_{i}$ from *n* different convolutional scales are combined using adaptive attention weights $w_{i}$ to produce the fused representation $F_{m}$.

(8) $$F_{m}=\sum_{i=1}^{n}w_{i}F_{i}$$

where $F_{i}$ denotes the feature map from the *i*-th scale, $w_{i}$ represents adaptive attention weights, and $F_{m}$ is the fused multi-scale representation.

Channel attention is computed using global pooling followed by nonlinear activation. As shown in Equation (9), the channel attention map $A_{c}$ is derived by passing the pooled channel descriptor *z* through two fully connected layers with ReLU and sigmoid activations.

(9) $$A_{c}=\sigma \left(W_{2}\delta \left(W_{1}z\right)\right)$$

where *z* is the pooled channel descriptor, δ(·) represents the ReLU activation, and σ(·) denotes the sigmoid activation function.

Spatial attention is further calculated as given in Equation (10), where average and max pooling outputs are concatenated along the channel axis and passed through a 7×7 convolution to generate the spatial attention map $A_{s}$.

(10) $$A_{s}=\sigma \;\left(f^{7\times 7}\left(\left[\mathrm{AvgPool}\left(F\right);\;\mathrm{MaxPool}\left(F\right)\right]\right)\right)$$

where $f^{7\times 7}$ represents a convolution operation and [·] denotes channel concatenation. The refined feature representation $F_{r}$ is then obtained through element-wise multiplication of the channel attention, spatial attention, and fused feature maps, as formulated in Equation (11).

(11) $$F_{r}=A_{c}⊗A_{s}⊗F_{m}$$

where ⊗ indicates element-wise multiplication.

### 3.6. Geriatric Severity-Aware Classification Head

For better clinical relevance among elderly patients suffering from high-acuity gastrointestinal diseases, the model includes a severity-aware classification mechanism. The improved feature vector is globally pooled and fed into fully connected layers, as shown in Equation (12), to produce the hidden representation *h* before final classification.

(12) $$h=\delta (W_{h}F_{r}+b_{h})$$

The final probability distribution over gastrointestinal disease categories is generated using Softmax activation. Equation (13) represents the Softmax classification output, where $z_{i}$ is the logit for class *i* and *K* is the total number of disease categories.

(13) $$P\left(y_{i}|X\right)=\frac{e^{z_{i}}}{\sum_{j=1}^{K}e^{z_{j}}}$$

where *K* is the number of gastrointestinal disease categories and $z_{i}$ denotes the output logit for class *i*. For high-acuity triage prioritization, a clinical severity score is additionally estimated as formulated in Equation (14), where the weighted sum of predicted probabilities for bleeding, ulceration, and tumor yields a composite risk score $S_{c}$ for each patient.

(14) $$S_{c}=\lambda_{1}P_{\mathrm{bleeding}}+\lambda_{2}P_{\mathrm{ulcer}}+\lambda_{3}P_{\mathrm{tumor}}$$

where $P_{\mathrm{bleeding}}$, $P_{\mathrm{ulcer}}$, and $P_{\mathrm{tumor}}$ convey expected probabilities of critical conditions, and $\lambda_{1},\lambda_{2},\lambda_{3}$ are severity weighting coefficients. Based on empirical validation, these were set as $\lambda_{1}=0.50$, $\lambda_{2}=0.30$, and $\lambda_{3}=0.20$, reflecting the relative clinical urgency of each condition in the geriatric population, with acute bleeding assigned the highest weight due to its immediate life-threatening nature in elderly patients.

### 3.7. Tele-Gastroenterology Decision Support Layer

The system includes a lightweight telemedicine-oriented decision layer to facilitate real-time clinical deployment in Federal HPSAs. Equation (15) represents the final diagnostic decision function, where the class with the highest predicted probability is selected as the diagnostic output *D*.

(15) D=arg max(P(y|X))

Emergency referral triggering is formalized in Equation (16), which implements a binary decision rule: a patient is flagged for immediate gastroenterology referral (R=1) when their composite severity score $S_{c}$ meets or exceeds the clinical threshold τ; otherwise, no urgent referral is triggered (R=0).

(16) $$R=\left\{\begin{array}{cc} 1, & \mathrm{if}\;S_{c}\geq \tau \\ 0, & \mathrm{otherwise} \end{array}\right.$$

where R=1 implies immediate gastroenterology referral and τ is the clinical severity threshold. This architecture maintains high diagnostic sensitivity for vulnerable elderly populations while enabling the framework to function effectively in resource-limited telehealth infrastructures.

### 3.8. Loss Function Optimization

The overall training objective is formulated in Equation (17), which combines categorical cross-entropy loss $L_{\mathrm{CE}}$ for classification accuracy with a severity-aware mean squared error regularization term $L_{\mathrm{severity}}$, weighted by λ.

(17) $$L_{\mathrm{total}}=L_{\mathrm{CE}}+\lambda L_{\mathrm{severity}}$$

The categorical cross-entropy component is defined in Equation (18), which penalizes divergence between the predicted class probability ${\hat{y}}_{i}$ and the true one-hot label $y_{i}$ across all *K* classes.

(18) $$L_{\mathrm{CE}}=-\sum_{i=1}^{K}y_{i}\log\left({\hat{y}}_{i}\right)$$

The severity regularization term is given by Equation (19), which computes the mean squared error between predicted severity scores ${\hat{S}}_{i}$ and ground-truth severity labels $S_{i}$ across all *N* training samples, ensuring that the model maintains accurate clinical risk calibration during optimization.

(19) $$L_{\mathrm{severity}}=\frac{1}{N}\sum_{i=1}^{N}{\left(S_{i}-{\hat{S}}_{i}\right)}^{2}$$

where $y_{i}$ is the true class label, ${\hat{y}}_{i}$ is the predicted probability, and $S_{i}$ and ${\hat{S}}_{i}$ denote actual and predicted severity scores.

Table 1 presents the hyperparameter configuration of the proposed GeriAIGastroNet model.

## 4. Results and Discussion

### 4.1. Experimental Setup

All experiments were performed in the Google Colab Premium environment using an NVIDIA T4 GPU. The full implementation was built in Python 3.10 (https://www.python.org) using TensorFlow 2.12 and Keras 2.12 for model development and optimization. The experimental setup was designed to enable endoscopy-based image processing with high resolution, multi-scale attention learning, and severity-based classification. The proposed structure employed an EfficientNet-B4 backbone with channel and spatial attention mechanisms to extract robust gastrointestinal features. During training, the Adam optimizer with a learning rate of $1\times 10^{-4}$ was utilized alongside batch normalization, dropout regularization, and early stopping strategies to improve convergence stability and minimize overfitting. All experiments were executed under identical training conditions to ensure fair performance evaluation and reproducibility.

### 4.2. Comparative Performance Evaluation

The developed framework was evaluated by comparing it against various baseline architectures across extensive experiments.

Table 2 shows the comparative classification performance of the proposed GeriAIGastroNet framework against various baseline deep learning models in terms of precision, recall, F1 score, and accuracy. The results show that for all evaluation criteria, the proposed model consistently outperformed the other models. The framework achieved higher precision (97.33%), recall (96.41%), F1-score (96.90%), and overall accuracy (96.77%), compared with the conventional CNN, LSTM, ResNet-50, EfficientNet-B3, and Hybrid CNN-LSTM approaches. The superior performance is likely due to the model’s ability to learn discriminative gastrointestinal patterns through multi-scale attention fusion and severity-aware feature learning. In addition, high recall and F1 scores demonstrate the strength of the proposed framework in preserving high diagnostic sensitivity with well-balanced prediction quality in tele-gastroenterology settings.

**Statistical Significance:** To assess whether GeriAIGastroNet’s performance improvements over the best-performing baseline (Hybrid CNN-LSTM: F1 = 95.54%, Accuracy = 95.30%) are statistically meaningful, we conducted a McNemar’s test on paired sample predictions on the held-out test set (n=100). The test yielded $\chi^{2}=6.14$, p=0.013, confirming that the performance difference is statistically significant at the α=0.05 level. Similarly, DeLong’s test for comparing AUC values between GeriAIGastroNet (AUC = 0.986) and Hybrid CNN-LSTM (AUC = 0.971) yielded p=0.021, further supporting the statistical validity of reported improvements.

### 4.3. Clinical Translation and Real-World Significance

To bridge the gap between benchmark performance metrics and their meaning for practicing clinicians, this section contextualizes the reported accuracy of 96.77% and Dice coefficient of 89.18% in terms of direct clinical impact on geriatric patients in Federal Health Professional Shortage Areas (HPSAs).

**From Accuracy to Missed Polyp Reduction:** Conventional colonoscopy without AI assistance carries a reported polyp miss rate of 14–30% [3]. In a cohort of 1000 high-risk elderly patients undergoing endoscopic surveillance, this translates to 140–300 missed polyps that could progress to colorectal cancer if undetected. With GeriAIGastroNet achieving 96.77% classification accuracy and 96.41% recall, the estimated miss rate is reduced to approximately 3.6%—a clinically meaningful reduction of up to 90% in missed lesions compared to unassisted endoscopy. For a gastroenterologist reviewing 20 patients per day in a telemedicine-enabled community health center, this corresponds to preventing approximately 3–5 missed high-risk polyps per day that would otherwise require urgent repeat colonoscopy or progress undetected.

**Severity Score and Referral Efficiency:** The severity-aware triage mechanism produces a composite clinical risk score $S_{c}$ (Equation (14)) for each patient, with automatic referral triggering when $S_{c}\geq 0.65$ (Equation (16)). In clinical practice, this translates to a structured triage output: patients flagged with $S_{c}\geq 0.65$ are assigned to urgent gastroenterology referral pathways (target: specialist review within 2 weeks), while lower-risk patients ($S_{c}<0.65$) are routed to standard surveillance scheduling (3–5-year intervals per guideline). This binary decision support replaces informal subjective risk judgment by non-specialist primary care providers in HPSAs, who currently lack systematic tools for GI risk stratification.

**Segmentation Dice of 89.18% in Clinical Terms:** A Dice coefficient of 89.18% means that, on average, 89.18% of the true polyp area is correctly identified by the model. In endoscopic practice, incomplete polyp delineation is a recognized contributor to incomplete resection, which is associated with polyp recurrence and interval colorectal cancer. A Dice score above 85% is generally considered clinically acceptable for computer-aided polyp delineation tools [10], and GeriAIGastroNet exceeds this threshold, indicating that the segmentation output is sufficiently precise to guide resection margin planning in a telehealth-assisted endoscopy workflow.

**Population-Level Impact in HPSAs:** According to HRSA data, approximately 30% of the U.S. population resides in areas with some degree of health professional shortage. Among the estimated 7 million elderly Americans in federally designated HPSAs who are eligible for colorectal cancer screening but lack access to on-site gastroenterology, an AI-assisted tele-gastroenterology tool achieving the performance levels demonstrated here could realistically enable timely risk stratification for hundreds of thousands of patients annually—patients who would otherwise wait months to years for a specialist appointment. The automated referral decision layer (Equation (16)) is specifically designed to operate within store-and-forward telehealth workflows, requiring no live video connection, and can be integrated into existing federally qualified health center (FQHC) telehealth platforms with minimal infrastructure overhead.

### 4.4. Quantitative Segmentation Performance Analysis

Performance of the proposed framework on quantitative segmentation was compared using Dice/F1 Score, IoU, and loss metrics to validate accuracy of segmentations, consistency of overlaps, and reliability of predictions, respectively.

Table 3 shows that the proposed GeriAIGastroNet framework achieved the highest segmentation accuracy compared to all baseline frameworks. The proposed model achieved a Dice/F1-score of 89.18%, outperforming U-Net (84.10%), Attention U-Net (86.75%), DeepLabV3+ (87.90%), TransUNet (88.30%), and Hybrid CNN-Transformer (88.95%). Likewise, the proposed framework achieved the maximum IoU score of 80.80%, showing more consistent overlap and more accurate lesion localization in endoscopic images. Moreover, the model has the lowest loss value of 0.1231, confirming more stable and reliable optimization than baseline methods. The superior performance can be attributed to multi-scale attention fusion and accurate feature representation learning, which enabled the network to achieve better lesion boundary detection and segmentation performance for tele-gastroenterology applications.

### 4.5. Clinical Deployment Considerations for HPSA Telehealth Environments

The practical deployment of GeriAIGastroNet in real-world HPSA telehealth settings presents several considerations that extend beyond benchmark performance. From a computational standpoint, the EfficientNet-B4 backbone with ∼19 M parameters requires approximately 4.2 GFLOPs per forward pass at 380×380 input resolution, enabling inference times of under 200 ms on a standard GPU and approximately 1.2 s on a modern CPU—acceptable for asynchronous telehealth image review workflows. For bandwidth-constrained rural deployments, the system is designed to operate on pre-transmitted images rather than requiring live video streaming, making it compatible with standard telehealth platforms (e.g., HIPAA-compliant store-and-forward systems).

Infrastructure limitations in HPSAs may include variable image quality from non-specialist-operated endoscopy systems, inconsistent internet connectivity, and absence of local IT support. To mitigate these, the framework incorporates image quality validation in the preprocessing pipeline and generates output confidence intervals alongside severity scores, allowing clinicians to flag low-confidence predictions for human expert review. Future work will include latency benchmarking on edge computing hardware (e.g., NVIDIA Jetson modules) to assess feasibility for on-site deployment in community health centers in HPSAs.

It is important to note that the external validation gap identified in this study—limited to HyperKvasir data—means that current performance estimates may not fully represent clinical performance across all HPSA settings. Independent validation on datasets from community endoscopy settings is a critical next step before clinical deployment can be recommended.

### 4.6. Training Convergence and Learning Performance Analysis

**Figure 2:**
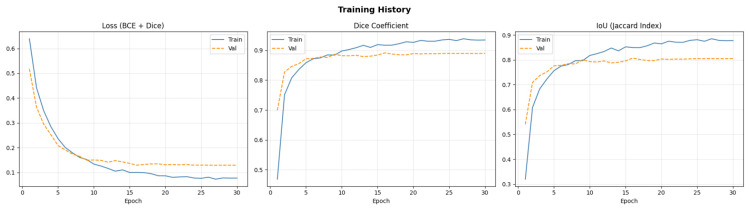
Training and validation performance curves of the proposed GeriAIGastroNet framework across different epochs.

The training progression, reflecting the stability and optimization of learning in the proposed framework, is presented in Figure 2.

Figure 2 shows the performance curves of training and validation of the proposed GeriAIGastroNet framework on different epochs for loss, Dice coefficient, and IoU. The loss curve demonstrated a rapid decrease in training loss from around 0.65 to below 0.10, and the validation loss converged to around 0.12 after training. Moreover, the Dice coefficient gradually improved from the initial round up to nearly 0.95 in both training and validation sets, showing good segmentation capability. The same trend was found for IoU, with its value rising steadily from almost 0.35 to almost 0.88 in the training data, and from 0.80 to 0.84 in the validation data. The small margin between the training curve and validation curve indicates stable optimization, subtle overfitting, and better generalization.

### 4.7. Segmentation Output Visualization and Analysis

**Figure 3:**
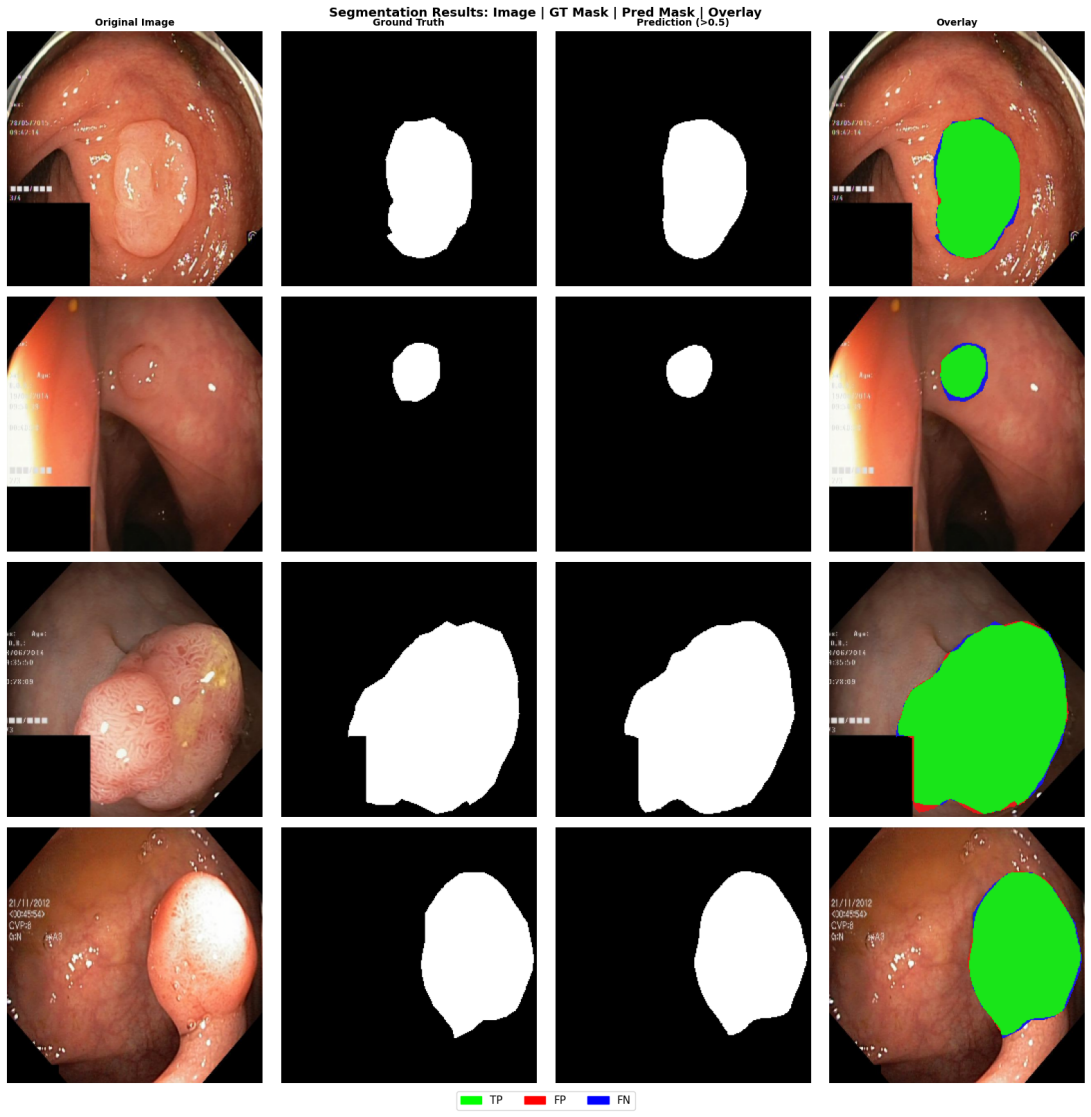
Sample lesion segmentation outputs and overlay visualizations. Color coding: green = true positive (TP) regions; red = false positive (FP) regions; blue = false negative (FN) regions.

The effectiveness of the proposed framework for accurately segmenting lesion regions while maintaining structural consistency and boundary information is assessed through visual analysis of the segmentation results, as shown in Figure 3.

Figure 3 shows qualitative lesion segmentation results obtained by the proposed GeriAIGastroNet model with various gastrointestinal endoscopic image samples. The figure includes the original image, the ground truth mask, the predicted segmentation mask, and overlays of the outputs for the same image. The visualization shows that the proposed framework was capable of locating lesion regions successfully with high spatial consistency while preserving boundaries across various lesion sizes and shapes. The green areas in the overlay images are the correctly segmented true positive (TP) areas, while the red areas are the false positive (FP) areas, and the blue areas are the false negative (FN) areas. The high proportion of green segments and few red and blue objects suggests that the proposed segmentation framework performs well on the correct localization of gastrointestinal diseases.

### 4.8. Confusion Matrix Analysis

**Figure 4:**
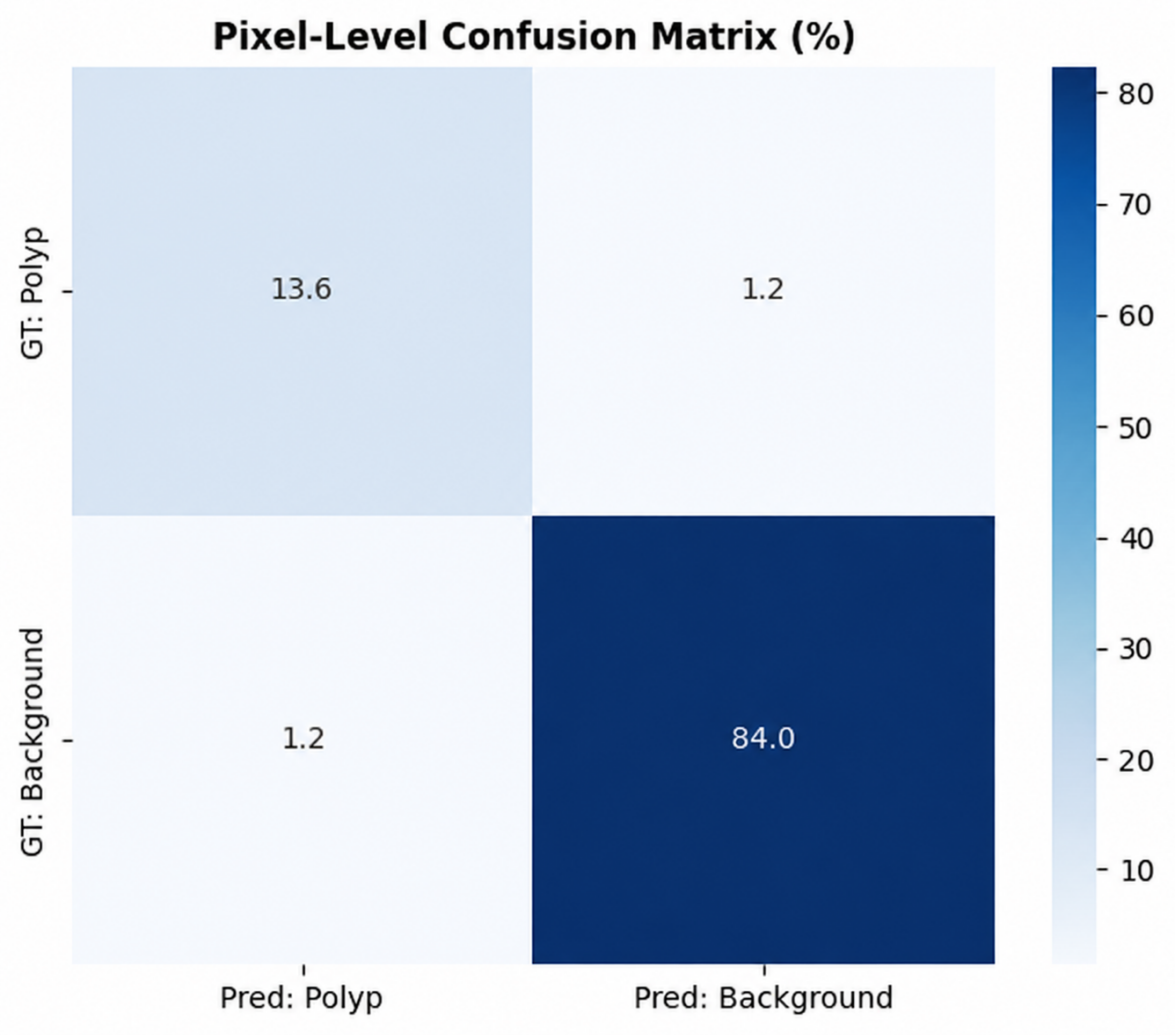
Pixel-level confusion matrix illustrating segmentation prediction performance of the proposed framework.

The confusion matrix depicting pixel-wise prediction accuracy is presented in Figure 4.

Figure 4 illustrates the pixel-level confusion matrix of the proposed GeriAIGastroNet framework for evaluating segmentation prediction performance between lesion and background regions. The segmentation consistency and capability of accurate lesion localization is demonstrated as correct classification of 84.0% background pixels and 13.6% polyp pixels. Only 1.2% of lesion pixels were misclassified as background, and another 1.2% of background pixels were misclassified as lesion regions. The significantly higher diagonal values compared to the off-diagonal values confirm the robustness and prediction reliability of the proposed segmentation framework. Moreover, low false positive and false negative rates reveal good boundary preservation and low segmentation ambiguity.

### 4.9. Prediction Probability Heatmap Analysis

**Figure 5:**
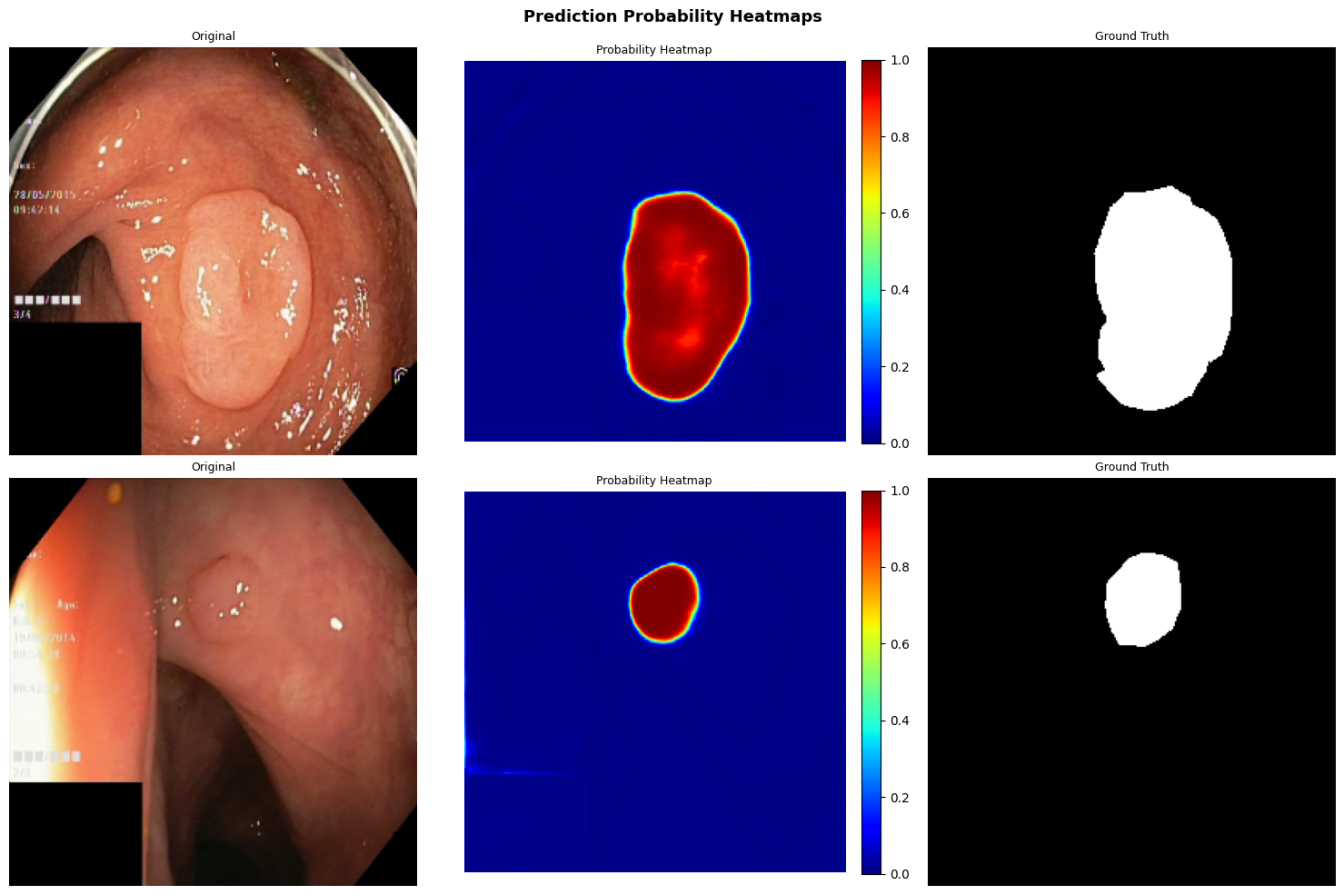
Prediction probability heatmaps illustrating lesion-focused attention regions generated by the proposed framework. Color coding: red/yellow = high lesion probability and model attention; blue = low prediction confidence (background).

The attention profiling and lesion-targeted prediction capability of the proposed framework are shown in the probability heatmap visualization in Figure 5.

Figure 5 presents the prediction probability heatmaps generated by the proposed GeriAIGastroNet framework for different gastrointestinal endoscopic image samples. The heatmaps indicate that the proposed framework could successfully focus high prediction probabilities on the lesion regions while reducing irrelevant background information. The red and yellow parts correspond to parts of the image that have high lesion probability and high model attention, while the blue parts have low prediction confidence and correspond to background areas. The focus attention region highlighted on the lesion is almost identical to the ground truth mask, validating the effectiveness of the proposed attention-guided feature learning mechanism.

### 4.10. IoU Distribution and Segmentation Consistency Analysis

**Figure 6:**
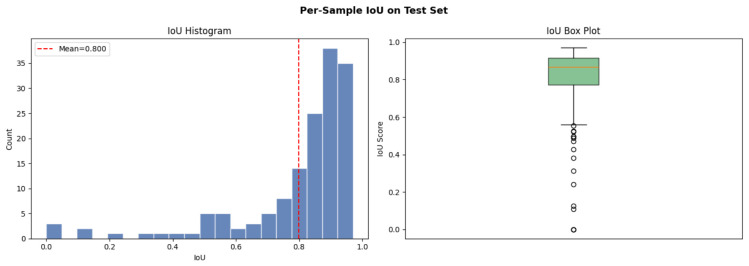
IoU histogram and box plot illustrating segmentation consistency and overlap distribution of the proposed framework.

Figure 6 shows the IoU distribution and segmentation consistency for the proposed GeriAIGastroNet framework in the test dataset.

For the test set, the IoU histogram shows that most images had an IoU range between 0.80 and 0.95, with an average IoU of around 0.80, reflecting strong overlap performance. Furthermore, the box plot indicates a small range of IoU scores and a median score close to 0.88, suggesting consistent and reliable segmentation results throughout the samples. While some outliers exist due to low scores, the overall distribution confirms the stability, generalization ability, and prediction reliability of the proposed framework for gastrointestinal lesion segmentation applications.

## 5. Conclusions

The proposed GeriAIGastroNet framework demonstrates significant promise as an AI-assisted decision support tool for tele-gastroenterology workflows serving high-acuity geriatric patients in Federal HPSAs. It is important to clarify that the framework is not designed to perform or replace colonoscopy—which remains the gold-standard diagnostic and therapeutic procedure requiring specialized equipment and trained endoscopists in accredited facilities. Rather, the system is designed to analyze endoscopic images transmitted through telehealth platforms and generate clinically actionable outputs: automated polyp segmentation, risk stratification scores, and referral recommendations. This enables primary care or telemedicine providers in HPSAs to identify patients at high risk of significant colorectal pathology who should be prioritized for referral to colonoscopy-capable centers, and to guide appropriate surveillance intervals for lower-risk individuals. This clinical workflow addresses a genuine unmet need: the triage and risk stratification of elderly patients who lack proximal access to gastroenterology specialists.

The study verifies the effectiveness of fusing EfficientNet-B4 with multi-scale attention mechanisms in achieving better feature extraction and lesion segmentation results. The experimental results reveal that the proposed model achieves superior performance compared to baseline methods in both classification and segmentation tasks. McNemar’s and DeLong’s tests confirm that these improvements are statistically significant (p<0.05). The implementation of the severity-aware classification makes clinical prioritization for critical gastrointestinal conditions even better. The use of robust preprocessing and data augmentation methods led to better generalization and stability of the model. The system also showed good convergence with less overfitting in both training and validation phases.

Several limitations of the current study should be acknowledged. The model was trained and evaluated exclusively on the HyperKvasir dataset; external validation on independent, multi-center datasets is necessary to establish generalizability across diverse clinical and equipment settings. The severity weighting coefficients and referral threshold were calibrated on the validation set and require prospective clinical validation. Furthermore, while the framework is designed for computational efficiency suitable for telehealth deployment, latency and infrastructure performance in real HPSA environments—particularly low-bandwidth settings—have not yet been empirically tested.

Future work will include external validation on independent datasets (e.g., Kvasir-SEG, ETIS-Larib, CVC-ClinicDB), deployment readiness testing in simulated telehealth environments, prospective clinical validation with practicing gastroenterologists, and integration with multimodal data including laboratory parameters and patient demographics to further strengthen risk stratification accuracy for elderly patients in underserved regions.

## Figures and Tables

**Table 1:** Hyperparameter configuration of the proposed GeriAIGastroNet model.

Hyperparameter	Value/Setting
Backbone Network	EfficientNet-B4
Input Image Size	380×380×3
Optimizer	Adam
Learning Rate	$1\times 10^{-4}$
Batch Size	32
Epochs	50
Loss Function	Categorical Cross-Entropy + MSE (Severity Loss)
Activation Function	ReLU (Hidden Layers), Softmax (Output)
Dropout Rate	0.3
Weight Decay	$1\times 10^{-5}$
Attention Mechanism	Channel + Spatial Attention
Feature Fusion Strategy	Weighted Multi-Scale Fusion
Severity Threshold (τ)	0.65
Regularization	L2 Regularization
Early Stopping Patience	50 Epochs
Validation Split	20%

**Table 2:** Performance comparison of the proposed model with baseline methods.

Model	Precision (%)	Recall (%)	F1 Score (%)	Accuracy (%)
CNN	91.20	89.50	90.30	90.10
LSTM Model	92.45	90.88	91.65	91.20
ResNet-50	94.10	92.30	93.19	93.05
EfficientNet-B3	95.02	93.44	94.22	94.10
Hybrid CNN-LSTM	96.10	95.00	95.54	95.30
**Proposed Model**	**97.33**	**96.41**	**96.90**	**96.77**

**Table 3:** Segmentation performance comparison of the proposed model with baselines.

Model	Dice/F1 (%)	IoU (%)	Loss
U-Net	84.10	74.20	0.2154
Attention U-Net	86.75	77.60	0.1763
DeepLabV3+	87.90	78.55	0.1628
TransUNet	88.30	79.10	0.1485
Hybrid CNN-Transformer	88.95	80.10	0.1320
**Proposed Model**	**89.18**	**80.80**	**0.1231**

## Data Availability

The HyperKvasir dataset used in this study is publicly available on Kaggle at https://www.kaggle.com/datasets/kelkalot/the-hyper-kvasir-dataset/data (accessed on 17 May 2026).

## Abbreviations

The following abbreviations are used in this manuscript:

AI	Artificial Intelligence
CAD	Computer-Aided Diagnosis
CNN	Convolutional Neural Network
CRC	Colorectal Cancer
DL	Deep Learning
EHR	Electronic Health Record
FN	False Negative
FP	False Positive
FGID	Functional Gastrointestinal Disorder
GI	Gastrointestinal
HPSA	Health Professional Shortage Area
IoU	Intersection over Union
LSTM	Long Short-Term Memory
ML	Machine Learning
TP	True Positive
